# Supplementary material for: Learning curve and surgical outcome of robotic assisted colorectal surgery with ERAS program
Source: Sci Rep. 2022 Nov 29;12:20566. doi: 10.1038/s41598-022-24665-w (PMC9709162; doi:10.1038/s41598-022-24665-w)
Supplement: Supplementary file 1 — Supplementary Information 1. [file 41598_2022_24665_MOESM1_ESM.zip › LSC Raw data-Ñ[▒K/101/H_ñw╝╨░O▒Kñσ.pdf]

正式摘要(電子簽章：000000202251538)<br>W52 / W52 ☐英文標題(注意:無英文姓名-請補病人基本檔!)	☐【讀取】憑證	

臺中榮民總醫院
出院病歷摘要

轉入號： 轉出號：

[redacted]

(8)流水編號	
(9)入院日期	109/10/28 (2020) 直腸外科 W52 - 112
(10)轉科日期	
(11)出院日期	109/11/02 (2020) 住院天數計 5 日

診斷

- 入院:1. Malignant tumor of T-colon
- 出院:1. Malignant tumor of T-colon, s/p laparoscopic right colectomy and anastomosis on 10/29
  2. CAD-II, s/p stent *6 (RCA proximal to middle ISR and LAD 1st DA proximal de novo lesion, post POBA+BMS for RCA lesion, POBA for LAD 1st DA lesion on 2012/04/30), under aspirin
  3. HTN, under valsarta and carvedilol
  4. BPH, s/p laser surgery

主訴

- Dizziness for 6 months

病史

- This 83 y/o male has past history of
  1. CAD-II, s/p stent *6 (RCA proximal to middle ISR and LAD 1st DA proximal de novo lesion, post POBA+BMS for RCA lesion, POBA for LAD 1st DA lesion on 2012/04/30), under aspirin
  2. HTN, under valsarta and carvedilol
  3. BPH, s/p laser surgery
  The patient lived in King-Men.
  This time, he suffered from dizziness for 6 months. Thus he went to department of family medicine where anemia was noted. Then he received colonscopy and colon cancer was suspected. According to his family's statement, there was no bloody nor tarry stool, bleeding tendence, diarrhea, constipation, poor appetite nor body weight loss. Abdominal CT revealed no obvious wall thickening or enhancing mass of the colon, nor definite mesocolon lymphadenopathy. Under the impression of colon cancer, he was admitted to our ward for pre-op preparation and further management.

體檢發現

- home BP 95-138/60-75
  no dyspnea or effort angina

  hypertneiosn follow up Kin Mon H. with aspirin, Valsarta, carvedilol 6.25 mh qd , aspirin and nitrate
  teh most recnet lab data, : hgb 12.8,glu 121 , chol 164, Cr 1/12HBA1C 5.6

  轉診院所：衛生福利部金門醫院
  - General appearance :
    - easy looking , conscious clear , without cardiopulmonary distress
    - Vital signs自動帶入date 體溫(BT) 脈搏(P) 呼吸(R) 血壓(BP) 身高(BH) 體重(BW) BMI
      20201028 35.6C 61/min 18/min 145/63mmHg 168cm 63kg 22.3
    - Vital signs: B.P. 145/63 mmHg , P.R. 61 /min , R.R. 18 /min , B.T. 35.6 ℃
  - HEENT :
    - Eyes: no anemic conjunctiva, no icteric sclera, normal EOM
    - ENT: no otorrhea, no rhinorrhea, no erythematous mucosa, no injected tonsil, no deformity

-
  - Neck: supple, freely movable, trachea midline,no deformity, no engorged jugular vein , no palpable thyroid or lymph node enlargement
  - Chest:
    - Inspection: Grossly normal, symmetric and fully expansion, no accessory muscle used, no operation scar, no spider angiomata, no gynecomastia
    - Palpation: no palpable mass or nodule, no subcutaneous emphysema, no tenderness
    - Ausculation: clear breathing sound
  - Heart:
    - Palpation: no thrill, no heave
    - Percussion: normal heart size
    - Ausculation: regular heart beat, normal S1, S2, no gallop, no murmur
  - Extremities: No deformity, full ranged of motion without limitation, no clubbing finger or nail cyanosis, no pitting edema, no ulcer or pigmentation, no obvious skin lesion, no varicose vein, no bone, joint or muscle tenderness

CRS CONDITION

- Karnofsky performance status: (90) Able to carry on normal activity; minor signs or symptoms of disease
- PE of Abdomen:
- CEA: 3.61 ng/ml
- Imaging Studies:
- Colonoscope report: One tumor at proximal T-colon, other one at distal T-colon, s/o tattooing and clip placement.
- Figures:
  1. colon & rectum

手術日期及方法

- 手術日期及方法20201029 1. Laparoscopic assisted subtotal colectomy with side to side anastomosis, GIA 80/3.8*2, Re-inforcement with silk: nil, air leak test: not done
  2. Vessels ligation of ileo-colic, right colic, middle colic artery, left colic and IMA
  3. Cut margin: proximal 18cm(colon), 5cm(terminal ileum), distal 15cm
  4. Adhesion barrier film(+)

住院治療經過

- After admission, operation was done on 10/29. Try clear liquid diet on 10/30. Try semiliquid diet on 10/31. Due to relative stable condition, MBD was permitted and OPD f/u is scheudled for further treatment.

合併症

一般檢查

特殊檢查

病理報告

其它

出院時情況

- 3.改門診治療

出院指示

藥名	劑量	劑量單位	用法	頻次	數量	數量單位	現況	天數	單位
Bokey cap 100mg	1	CAP	PO	QD	14	CAP	TH	14	天
Pantoloc tab 40mg	1	TAB	PO	QDAC	14	TAB	TH	14	天
Ultracet tab	1	TAB	PO	Q6H	56	TAB	TH	14	天
OCAS Harnalidge 0.4 mg	1	TAB	PO	QDAC	14	TAB	TH	14	天

- Mag. Oxide tab | 2 | TAB | PO | TID | 84 | TAB | TH | 14 | 天

出院計劃

主治醫師 2062F 蔣鋒帆 / 住院醫師 2310K 陳璿夫

正式摘要**(**電子簽章：**000000202251528)**

W52 / W52

doctorRank:B
